# Two minds, one body: neural correlates of processing of We-information during shared control over a humanoid robot

**DOI:** 10.1098/rstb.2025.0163

**Published:** 2026-11-10

**Authors:** Mateusz Woźniak, Romina Haxhi, Davide De Tommaso, Agnieszka Wykowska

**Affiliations:** ^1^ Social Cognition in Human-Robot Interaction Unit, Italian Institute of Technology, Genoa, Italy; ^2^ Institute of Applied Psychology, Jagiellonian University, Cracow, Poland; ^3^ Hans-Fischer Senior Fellow, Institute for Advanced Study, Technical University of Munich, Lichtenbergstrasse 2 a, D-85748 Garching, Germany

**Keywords:** self, We-information, shared control, embodied self, social self

## Abstract

When speaking about the self, we typically think about ‘I’ or ‘Me’. However, our relations with other people are also a critically important aspect of our self, forming our ‘We’. In the current study, we investigated processing of information about the We during joint control over a single body. While naturally such a case occurs among the conjoint twins, we reconstructed such a situation by allowing two people to share control over a single humanoid robot. Using EEG, we investigated which neurocognitive processes are affected by providing We-information, i.e. information that conveys knowledge about properties at the group-level, while being uninformative about the individuals. We found that processing of We-information engages a cascade of processes, as indexed by various event-related potentials (ERPs), involving perception (P1, N1 and P2p), action selection (N2), working memory (P3b) and response *preparation* (contingent negative variation, CNV). We-information also modulates perception and processing of the subsequent information about the robot's target position, as indicated by modulation of similar ERPs (P1, N2 and P3b). Moreover, we found that We-information can affect dynamic allocation of covert attention towards the side of the partner controlling the other side of the robot. Overall, our results show that processing of information about the We can have a widespread influence on cognition, similarly to processing information about the self.

This article is part of the discussion meeting issue ‘From being to identity: analysis and synthesis of the self’.

## 1. Introduction

Throughout history, a single human mind has typically occupied a single human body, with rare exceptions such as conjoined twins. Dicephalic parapagus twins, for example, have two distinct heads sharing a single body [1,2]. These individuals must learn to coordinate with each other to jointly control their shared body. These rare natural cases illustrate that bodily self can, under certain conditions, extend beyond the boundaries of a single individual's body and reach into the social domain. The social self is typically understood as encompassing conceptual and seemingly disembodied aspects, such as social or group identities [3–6]. However, cases of shared bodily control illustrate that the social self can also take profoundly embodied forms: one's sense of self may extend beyond one's own body and become intertwined with another's. While conjoined twins represent an extremely rare natural occurrence, contemporary technologies—such as virtual reality systems or robotic interfaces—create novel contexts in which shared bodily control between people, or between humans and artificial agents, becomes possible. These scenarios allow us to experience, at least temporarily, how deeply social and bodily dimensions of the self can merge.

Sharing bodily control with another (artificial or natural) agent requires one to step beyond the ‘Me’ of one's self, and to embrace and integrate the ‘You’ and ‘We’. Research in joint action has long studied how individuals coordinate to achieve shared goals [7,8], and recent work has focused on how we can experience and represent the ‘We’ [9–12]. These proposals frequently distinguish between self- and other-representations, marking information related to individual agents, and We-representations, which refer to information related to the group level. We-representations are hypothetical internal mental constructs and cannot be directly observed. What can be manipulated, however, is We-information, which refers to information that can be provided by the environment (or experimental instructions or task) about properties at the group-level. Moreover, ***pure*** We-information relates to content that provides information about We (for example, about a relational structure between the individuals involved in a joint action), while being uninformative about the properties of the involved individuals. As such, using it allows the presentation of group-level information, which is dissociated from individual-level information. Throughout the rest of the text, we will refer to pure We-information whenever using the term We-information. Finally, We-information can be understood as a specific type of content that allows us to successfully cooperate with each other, and as such it can be understood as one of the tools allowing us shared intentionality [13–16].

Kourtis *et al.* [17] empirically investigated processing of We-information in the context of a task in which two people were acting together. In such a context, We-information can provide information about the relational structure between actions of partners, in contrast to information about individual movements. The authors used a cueing paradigm in which pairs of participants had to raise their hands together and form ‘structures’ involving palms of the hands facing either in the same or a different direction. When doing the task, participants first saw a cue that could provide information about what their individual action would be. Crucially, it could also provide We-information, indicating whether the relationship between their and their partner's action would be: the same (both partners raising their hands in the same position) or different. They found that providing We-information could speed up participants’ reaction times and increase synchrony between their actions, regardless of whether information about their individual actions was provided or not. Moreover, they found that providing We-information affected participants’ brain activity at several stages. Specifically, they found that it led to the increased amplitude of the P600 component, which has been shown to represent processing of structured representations in multiple domains [18,19], and an increased amplitude of the contingent negative variation (CNV), which is involved in decisional and action anticipation/preparation processes [20,21]. These results have shown that in the context of interpersonal coordination, We-information can be reflected in neural activity and can affect neural correlates of action preparation.

Hitherto, research on the processing of We-information has focused on situations when two individuals coordinate their respective bodies in an interpersonal scenario. However, the topic of how it is processed when two individuals jointly control a single body remains largely unexplored. One exception is a recent behavioural study by Woźniak *et al.* [22], in which participants engaged in joint control over a single humanoid robot body, using the iCub robot [23]. Participants were seated behind the robot and were told to jointly teleoperate it by controlling one of its arms, with the participant on the left controlling the left arm of the robot using their left arm, and *vice versa* for the participant sitting on the right. The task was to reproduce the robot position shown to participants on the screen. Importantly, before being shown the target position, participants could also receive We-information specifying whether each arm would perform the same or different movements from the other arm. This study partially replicated the findings from [17] and found that We-representations can lead to enhanced performance (faster reaction times (RTs)), but only if the robot movement was the same for both operators. This suggests that if movements performed by two people are incongruent, this might lead to a conflict and subsequent disappearance of the beneficial effect of the We-information. Moreover, this study did not find evidence that We-information increases behavioural synchrony between people. It did, however, find that participants reported strongly increased reported self–other overlap with both the robot and the other participant, as measured using the inclusion of other in the self (IOS) scale [24], suggesting that such joint bodily control can lead to some form of integration of both the robot and partner into one's self-representation. What remains unclear, however, is how these effects are reflected in neural activity, and whether such activity mirrors effects observed in dyadic interactions.

### (a) The current study

Building on [22], the present study investigated the neural processes involved in processing We-information during embodied joint control over a humanoid robotic body. Our study addressed two questions: (i) which neural processes are engaged when participants process We-information? and (ii) how does We-information influence recognition and execution of joint actions? To answer these questions, we replicated the previous behavioural set-up [22] with dual EEG recordings and a slightly modified set-up (participants switched sides and which robot arms they controlled only once, midway through the experiment, rather than after each block).

We based our experimental predictions partially on the study by Kourtis *et al.* [17], which used EEG to investigate processing of We-information during interpersonal coordination, and found evidence of modulation of the P600 and CNV components. Hence, we expected to observe modulation of these two components. However, by contrast to that previous study, we decided to collect a much larger sample (48 participants instead of 24), allowing us also to detect weaker effects. Therefore, our predictions involved a wider group of ERP components.

Specifically, for answering question (i), we wanted to evaluate whether processing We-information affects several of the most widely investigated event-related potentials (ERPs) involved in visual perception, working memory and response preparation. To test at what stage of visual perception We-information affects cognition, we investigated three posterior components: the P1, N1 and P2p, linked to progressively more advanced stages of visual processing. In regard to evaluating the early stages of response preparation, we hypothesized that if We-information affects it, then we should observe modulation of the frontal N2 [25]. Further, we expected that if processing of We-information involves the updating of working memory then we should observe its effect on the P3b [26]. Finally, we expected to replicate the findings from [17] and observe an increased amplitude of the P600 and CNV when We-information is presented. In regard to question (ii), we aimed to answer it by performing analyses time-locked to the go-signal that involved the complete information about the movements to be performed by both participants. We expected that earlier presentation of We-information might modulate how that full information is perceived and used for response preparation. We hypothesized modulation of the same components as above, with the exception of the CNV because the go signal was immediately followed by the participant's response, so no preparatory activity was required to observe the CNV.

Moreover, for both research questions, we were interested in determining whether We-information affects participants’ covert spatial attention [27] towards their partner's side. This can be assessed through the magnitude of the family of contralateral posterior lateralized components, known as the N1pc, N2pc or posterior contralateral negativity (PCN) [28,29].

Overall, our goal was to investigate which stages of neurocognitive processing are affected by presentation of the We-information, and whether they are also sensitive to the type of presented We-information (same versus different) within the context of joint control over a single robotic body. Our paradigm differs from classic joint action paradigms in which individuals coordinate separate bodies towards a shared goal. In our study, two agents jointly controlled one robot, creating a situation in which coordination is embodied within a single artificial agent.

## 2. Methods

### (a) Participants

Twenty-four pairs took part in the experiment, amounting to 48 individual participants. Of these, 31 were female and 17 were male. There were nine female pairs, two male pairs and 13 mixed pairs. The average age of participants was 27.9 years (s.d. = 10.3, median = 24, range = 20–63). Forty-one of the participants were born right-handed and seven left-handed. There were 17 pairs of two right-handed participants and seven mixed pairs.

The sample size was chosen *a priori* before the start of data collection to include 48 individual participants, which amounts to 24 pairs. The sensitivity analysis conducted using G*Power v.3.1.9.7 [30] for a repeated measures ANOVA with eight measurements (alpha = 0.05 and 1-beta = 0.8) revealed that we should be able to detect the effect size of *f* = 0.14 for participant-level analyses and *f* = 0.19 for pair-level analyses.

All participants gave written informed consent after reading the general information about the study and before starting the experiment. The study was approved by the local ethical committee (Comitato Etico Regione Liguria). The data were collected at the Italian Institute of Technology, IIT in Genoa, Italy, in accordance with the standards from the Declaration of Helsinki.

### (b) Design

The experiment comprised a 2 (We-information: present versus absent) × 2 (robot configuration: same versus different) repeated-measures design. The main dependent variables were mean amplitude of selected ERP components, as well as reaction times (for individual participants) and response asynchrony (for pairs). For EEG analyses that involved activity from the lateral electrodes (P1, N1 and P2p), we added the hemisphere (left versus right) as an additional factor to control for lateralization of the results. For the cue-locked analyses, the factor robot configuration was nested in trials with We-information present, so rather than an ANOVA, within-subject *t-*tests were used independently for the We-information and robot configuration factors. In these cases, ANOVA was used only for ERP analyses that involved hemisphere as a factor (P1, N1 and P2p). In all ANOVAs, significant interactions were followed with *post hoc* tests with Holm correction.

### (c) Procedure

#### (i) Experimental task

The participants were instructed to perform movements by controlling the iCub's arms. At the beginning of each movement, the robot's arms were bent at the elbows and pointed forward in what was called the ‘home’ position. Each arm could be moved to one of two target positions: an ‘up’ position where the arm extended sideways and slightly upward, or a ‘down’ position where the arm pointed downward (figure 1A). These movements were identical for the robot's left and right arms. Since each arm had two possible target positions, overall there were four arm configurations: (a) both arms up, (b) both arms down, (c) left arm up with right arm down, and (d) right arm up with left arm down. These configurations formed two categories: **same**, when both arms were moved to the same position (a and b), and **different**, when each arm reached a different position (c and d).

**Figure 1.**
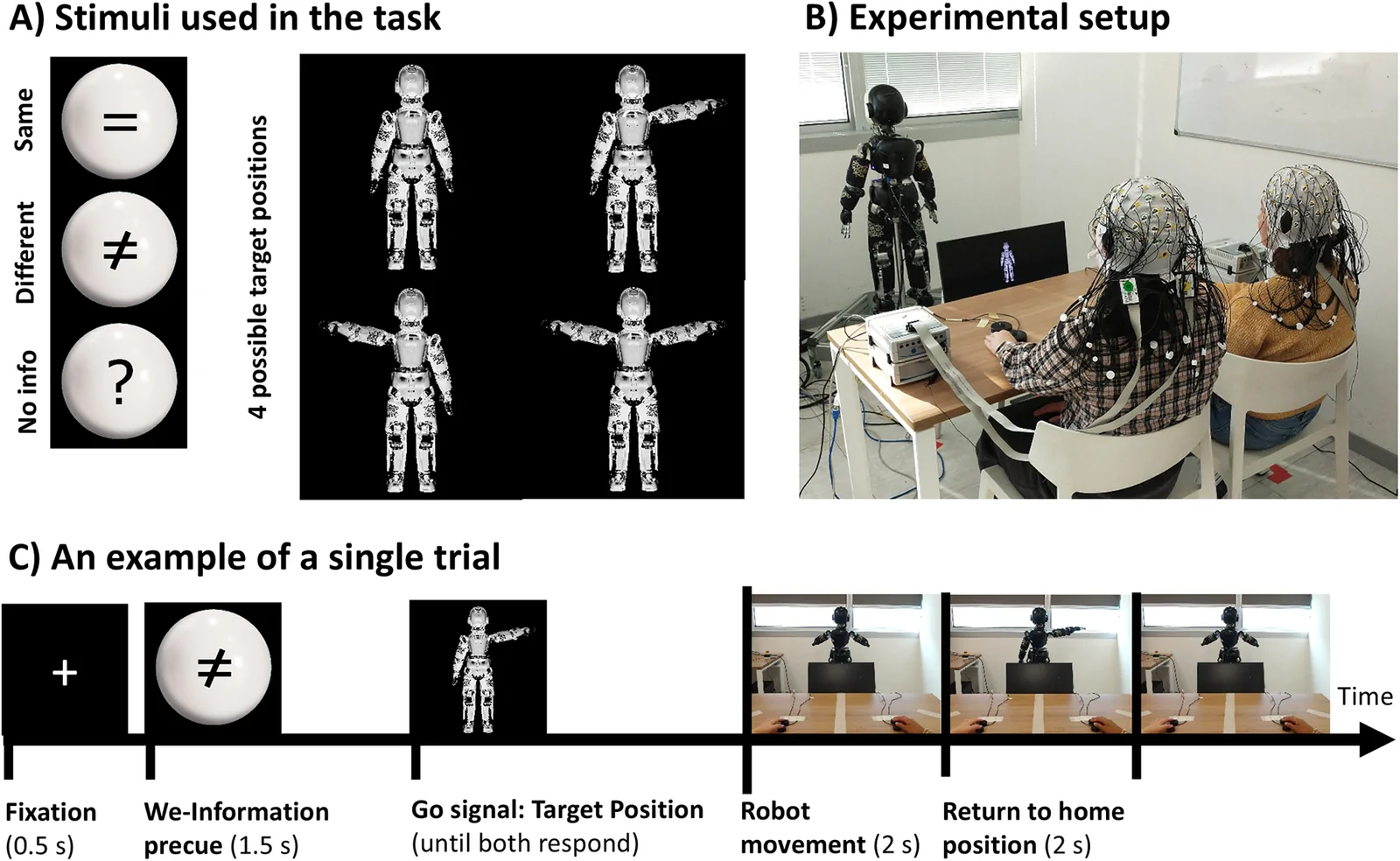
The procedure of the experiment.

Participants operated the robot in pairs. They sat at a table positioned behind the robot, with one person seated on the left side and the other on the right. Each person was responsible for controlling one of the robot's arms, which always corresponded to their seating position: the participant on the left managed the robot's left arm, while the one on the right controlled the right arm. In addition, participants always used the hand that matched the robot arm they controlled. This meant that the person on the left used their left hand to operate the robot's left arm, and the person on the right used their right hand to control the robot's right arm.

Each trial began with a We-information cue, which either indicated the target robot arm configuration or gave no information at all. This cue was shown for 1500 milliseconds and could appear in one of three forms (figure 1A):

— An equal sign, which signalled that both participants would perform the same movement (both arms either up or down). This appeared in 25% of the trials and resulted in both types of ‘same’ arm positions equally often.
— An unequal sign, which indicated that each participant would perform a different movement (one arm up and the other down). This was also presented in 25% of the trials and produced both types of ‘different’ positions equally frequently.
— A question mark, which gave no hint about the target configuration. This was displayed in 50% of the trials and resulted in all four possible arm positions being equally likely.

After the We-information cue, the target position (go signal) was shown. This was displayed as a black-and-white negative image of the iCub robot viewed from behind (figure 1A). The target position remained visible on the screen until both participants pressed their respective keys to indicate how they wanted the robot arm they controlled to move. This action triggered the robot's movement, which lasted about 2 seconds and was then immediately followed by the arms returning to the ‘home’ position (see the supplementary video for an example).

**Figure 2.**
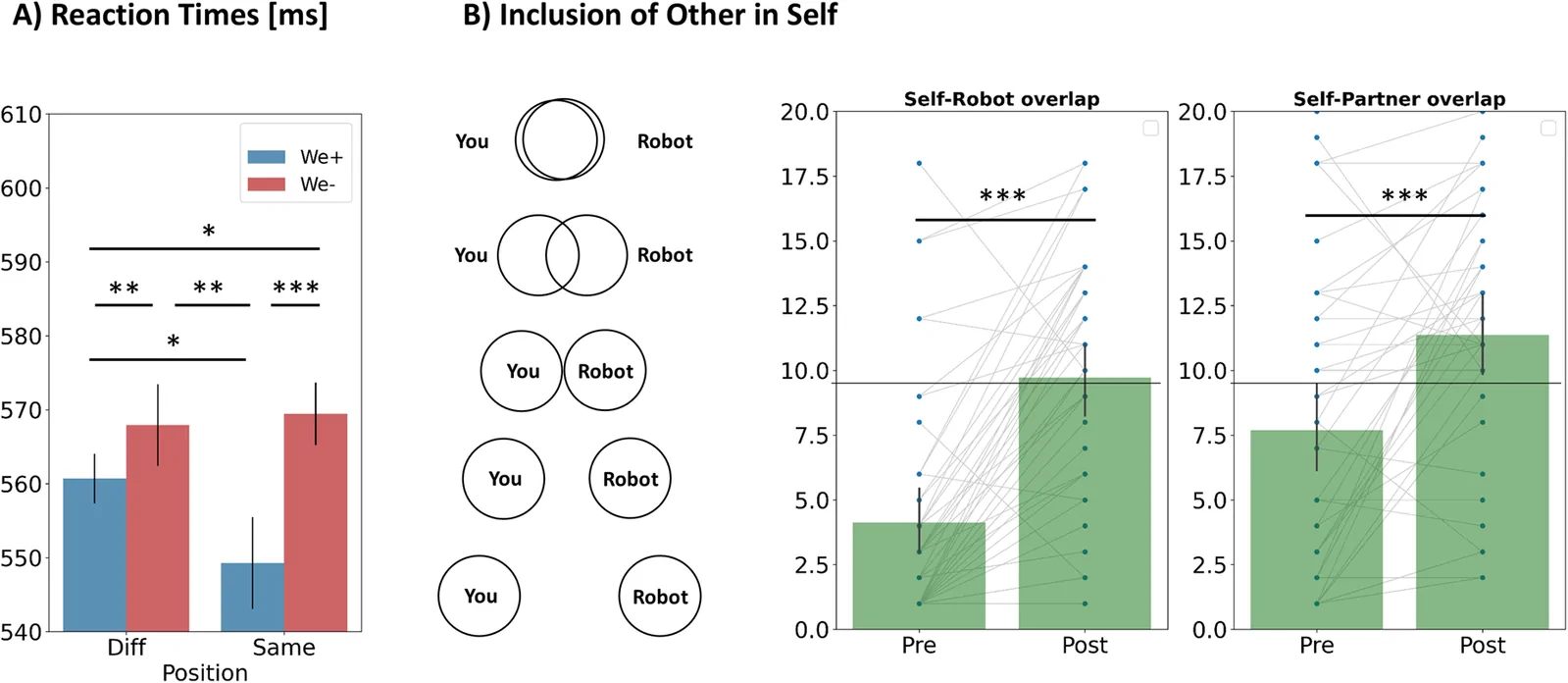
Behavioural results of the study. Error bars indicate 95% CIs. * p<0.05, ** p<0.01, *** p<0.001

**Figure 3.**
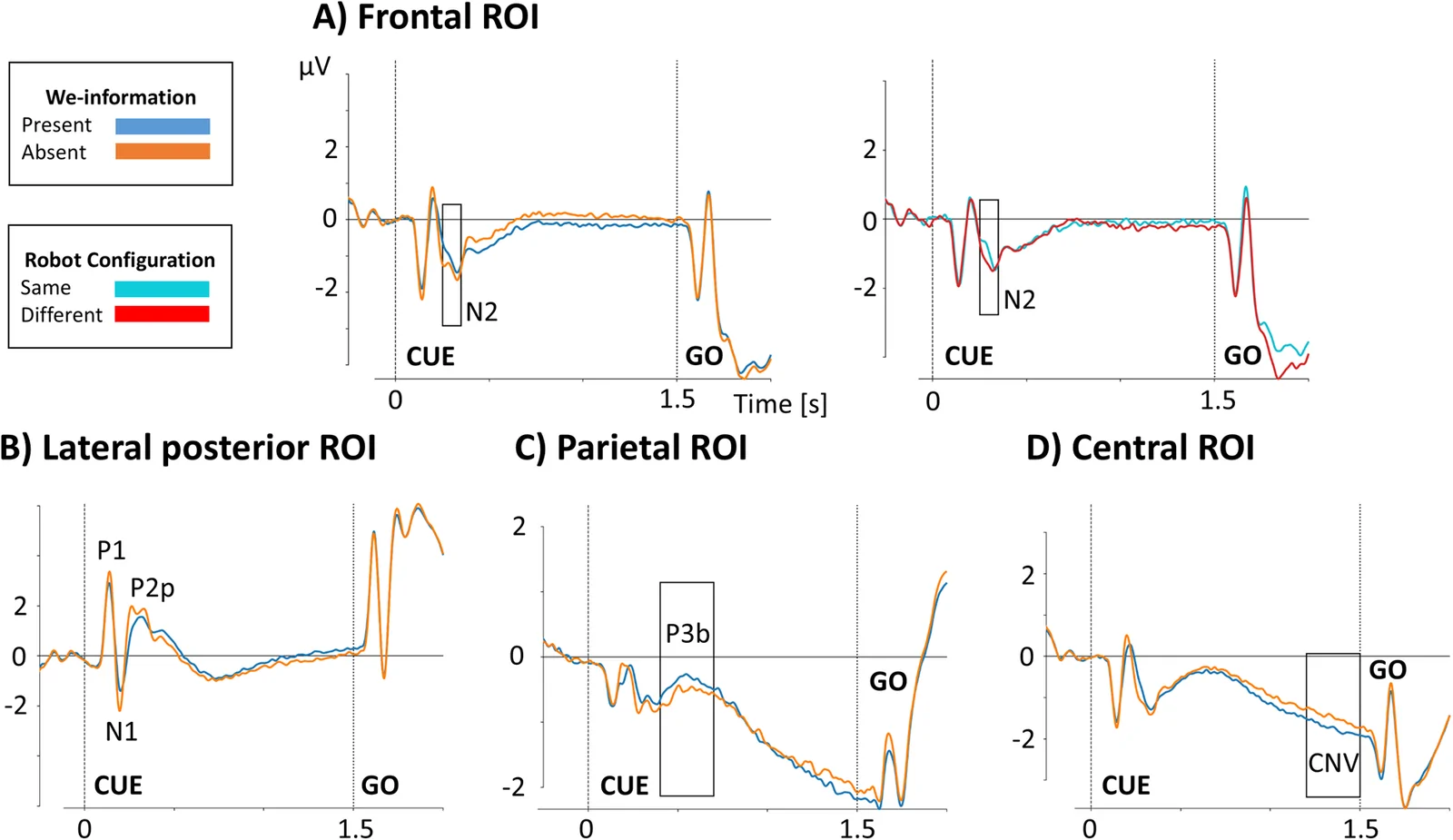
Results of the cue-locked analyses reflecting processing of the We-information. ROI, region of interest.

**Figure 4.**
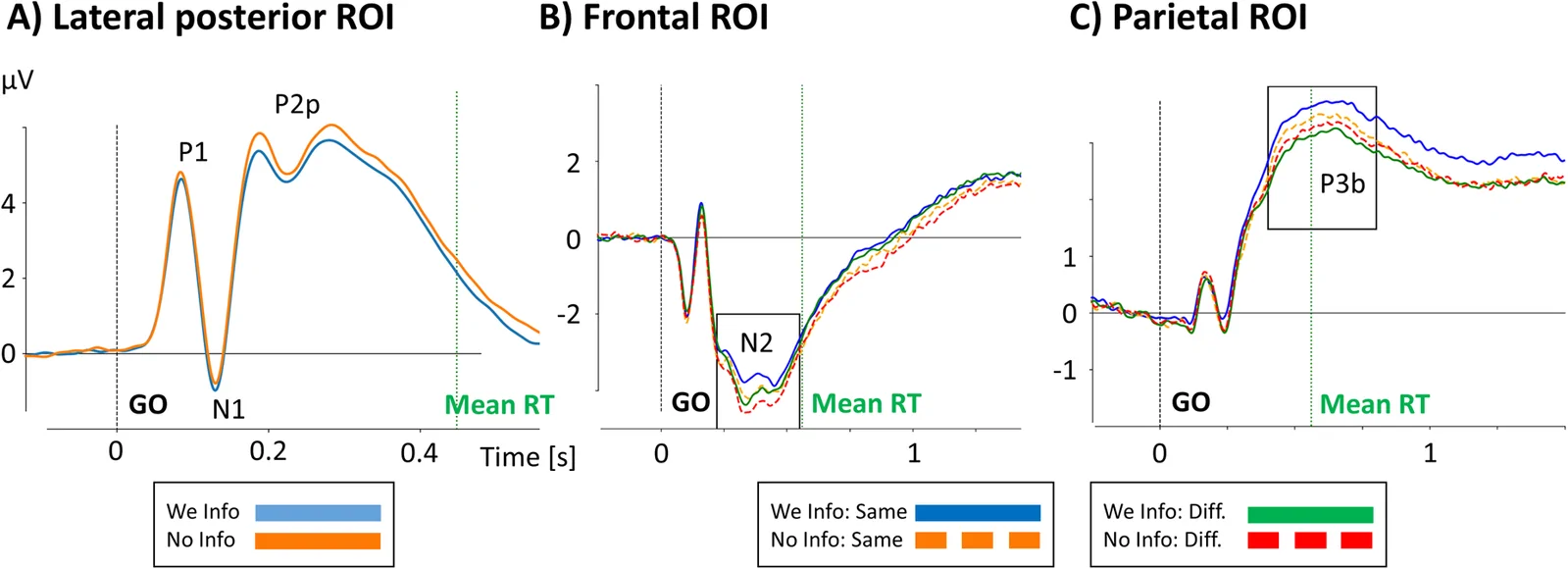
Results of the go-signal-locked analyses reflecting how We-information affects processing of the robot's target position, as well as action preparation and evaluation. Figure 4A shows only the comparison between We-information present and absent, averaged for both hemispheres. ROI, region of interest.

**Figure 5.**
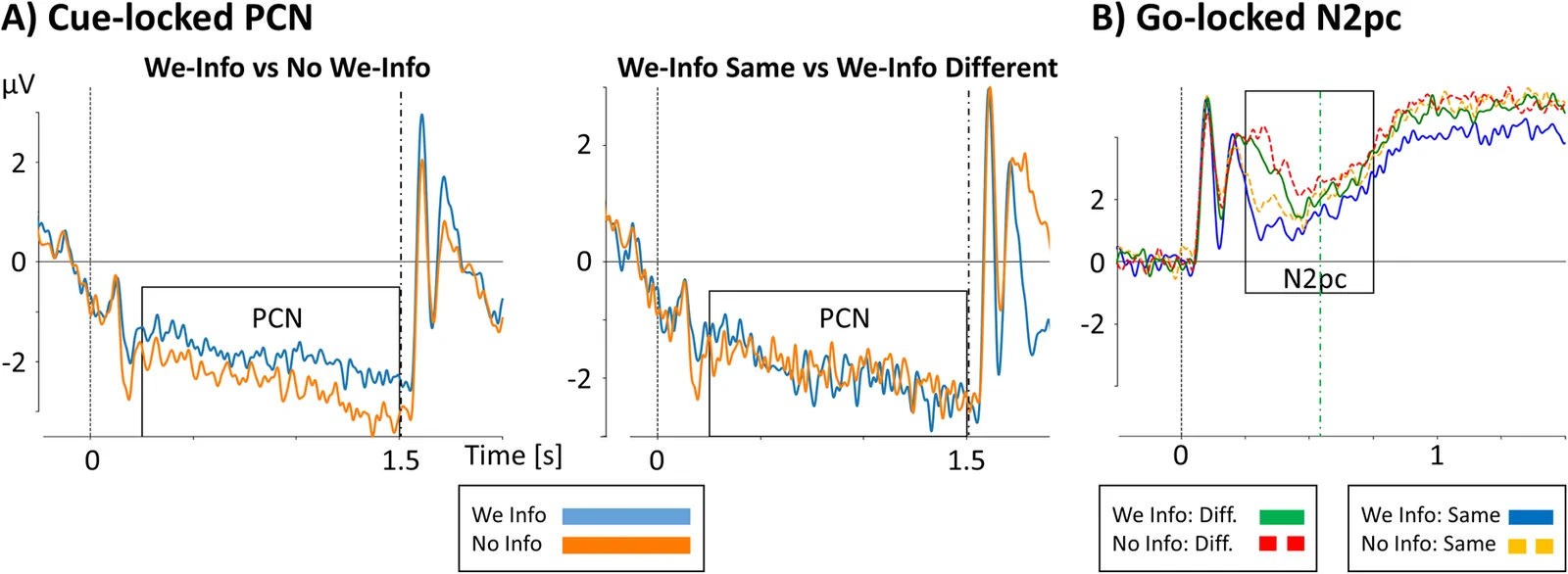
Illustration of the PCN (Cue-locked) and the N2pc (Go-signal-locked).

Each pair began with a practice block of 24 trials during which the experimenters explained the task. Afterwards, participants completed 10 experimental blocks, each containing 48 trials, for a total of 480 experimental trials used in the analysis. After the first five experimental blocks, participants swapped seats so that the person who had controlled the left arm would switch to the right arm, and *vice versa* (owing to technical issues at the beginning of data collection, the first pair swapped places after six blocks, followed by four blocks with the reversed seating, and the data from the second pair were collected from nine blocks among which four blocks involved the reversed seating).

At both the start and the end of the experiment, each participant completed two versions of the IOS scale [24,31] on paper. The IOS scale consists of a series of Venn diagram-like images showing circles that overlap to varying degrees, symbolizing the relationship between the participant and another person or entity. Participants selected the diagram that best illustrated how close they felt to the given target using a 20-point scale. We employed the version of the IOS scale in which the maximal distance is reflected by two circles separated by the distance of one circle's diagonal. In this study, participants completed IOS for both the robot (‘How strongly did you feel that the robot is a part of you?’) and their experimental partner (‘How strongly did you feel that the other participant is a part of you?’). The responses to the IOS were given on paper.

### (d) Apparatus and stimuli

In this study, we used the iCub robot, which has 53 actuated degrees of freedom and was developed by the Italian Institute of Technology [23]. This flexible, open-source robot is widely used in human–robot interaction research worldwide, thanks to its humanoid form, rich set of open-source resources, compatibility with popular development platforms and extensive options for customization. These features enabled us to efficiently program the robot's required movements for our experiments.

The robot's movements were programmed and carried out using the Yet Another Robot Platform (YARP) middleware [32]. Specifically, we employed YARP's IPositionController, a position controller designed to create joint trajectories that follow a minimum jerk profile. The task required precise coordination of the robot's left and right arm movements. To maintain symmetry between movements to the right and left, we first programmed the motion in one direction and then mirrored the joint trajectories for the other side. Video demonstrations of these movements are provided in the supplementary material.

Images of the robot's target positions were captured using a Sony camera in full HD resolution and then edited with ClipStudio Paint software [33]. The experiment was carried out on a PC running the Ubuntu operating system [34], and it was designed and executed using Psychopy v. 2022.2.4 [35].

### (e) EEG data acquisition

EEG was recorded continuously throughout the experiment independently for each participant. The EEG set-up for each participant involved a 10–20 cap (EasyCap, Brain Products GmbH, Germany) with 64 active electrodes relative to left mastoid reference. Electrode impedance was kept below 10 kΩ. EEG signals were amplified using a band-pass of 0–250 Hz by two actiCHamp amplifiers (Brain Products GmbH, Germany) and sampled at 500 Hz.

### (f) Data processing and analysis

Behavioural data analysis was performed using custom Python scripts and JASP [36]. For reaction time, data trials with values exceeding 2.5 standard deviations above each participant's mean were excluded from analysis. Reaction time asynchronies were calculated for each group as the average of the absolute difference between RTs of both participants.

Processing and analysis of the EEG data were performed offline using custom scripts written using MNE [37], a Python toolbox for EEG data analysis. First, we discarded the data from electrodes AF7, AF8, FT9, FT10, FT7, FT8, T7, T8, TP7 and TP8. We decided to discard these channels because they are very sensitive to various types of EEG noise (muscle activity and drift caused by sweat etc.), and activity in these electrodes was not of our theoretical interest. This allowed us to retain a larger number of valid trials, which was especially important for some of our analyses that required long time windows for segmentation. After discarding these channels, we re-referenced the data to a grand average from all remaining electrodes. Afterwards, we performed independent component analysis (ICA) to remove oculomotor and muscle artefacts. The ICA decomposition was performed on a copy of the data that was high-pass filtered at 1Hz and low-pass filtered at 100 Hz. We extracted 52 components using the fastICA algorithm (max_iter parameter set to ‘auto’). When performing ICA decomposition, the data recorded between the experimental blocks were excluded (reject_by_annotation = True). After selecting the components for exclusion (representing eye movements or muscle activity; average excluded components = 2.52), we performed the inverse ICA to obtain the reconstructed raw data.

For the ERP analyses, we first performed high-pass filtering at 0.1 Hz and low-pass filtering at 30 Hz to remove motor noise (for two participants, we used high-pass filtering of 1 Hz to remove strong drift from the data). For the cue-locked analyses, we segmented the data using the time window starting 500 ms before and ending 2000 ms after the onset of the cue (meaning that it also ended 500 ms after the onset of the go-signal), and baseline corrected in relation to the last 200 ms before the onset of the cue. For the go-signal-locked analyses, we segmented the data from –500 to 2000 ms, and performed baseline correction in relation to the last 200 ms before the go-signal. During segmentation, trials in which at least one electrode exceeded the min–max criterion of 120 μV (150 μV in case of one participant) were removed, leading to rejection of on average between 3.0 and 3.3% trials in total (s.d. = 2.85–2.96), depending on the time-locking of segmentation.

The ERP analyses were conducted for four preselected regions of interest (ROIs) that were chosen to allow us to investigate the main ERPs of our theoretical interest. We included the left and right lateral posterior ROI (left electrodes: PO7, P7, P5; right electrodes: PO8, P8, P6), where we expected to observe modulation of early visual components: P1, N1 [28] (references indicate studies with similar choice of electrodes) and posterior P2 [38]; the frontal ROI (AFz, Fz, FCz, F1, F2) for the N2 component [39]; the parietal ROI (CPz, Pz, CP1, CP2) to observe the P3b [40]; and the central ROI (FCz, Cz, CPz, C1, C2) to observe CNV [41]. The lateralized components (N1pc, N2pc, PCN) were calculated using the standard procedure involving double subtraction method and electrodes PO7 and PO8 [42]. The lateralization was calculated with respect to the location of the other participant, i.e. so increased negativity of the N2pc reflected enhanced attention to the side of the other participant. Importantly, the assignment of partner's side versus participant's own side was counterbalanced across the experiment by having participants swap seating positions halfway through the session, ensuring that lateralized effects could not be attributed to fixed hemispheric or spatial biases.

The timing of each component was estimated based on the inspection of the aggregate grand averages of the signal at the group level, as advocated by Brooks *et al.* [43]. The timings of each component were as follows. For the Cue-locked analyses: lateral posterior electrodes showed a P1 between 0.12 and 0.16 s, an N1 between 0.18 and 0.22 s and a posterior P2 (P2p) between 0.25 and 0.35 s. At the frontal sites, an N1a was observed between 0.12 and 0.16 s, a P2 observed between 0.18 and 0.22 s and an N2 between 0.25 and 0.29 s. At the parietal sites, a P3b appeared between 0.4 and 0.7 s and on the central sites, contingent negative variation was observed between 1.2 and 1.5 s. For the Go-locked analyses: at the lateral posterior sites, P1 (P1p) was identified between 0.09 and 0.12 s, N1 was identified between 0.15 and 0.18 s and P2p was exhibited between 0.22 and 0.27 s. At the frontal sites, N1a appeared between 0.08 and 0.12 s, a P2 was observed at 0.14–0.18 s and N2 identified between 0.22 and 0.26 s, while the electrodes on the parietal sites showed P3b between 0.4 and 0.8 s. For the lateralized components analyses included a cue-locked N1pc between 0.16 and 0.22 s and a sustained PCN between 0.25 and 1.5 s, as well as a go-locked N1pc between 0.14 and 0.17 s and an N2pc between 0.25 and 0.50 s.

## 3. Results

### (a) Behavioural results

#### (i) Accuracy, reaction times and asynchrony

The accuracy in the task was at the ceiling level in all experimental conditions, reaching the average level of 99.5% overall.

A 2 × 2 ANOVA of reaction times revealed a significant main effect of We-information (*F*(1,47) = 40.8, *p* < 0.001, partial *η*^2^ = 0.47), reflecting faster RTs when We-information was present. The main effect of configuration (same versus different) was not significant (*F*(1,47) = 2.1, *p* = 0.154), but the interaction was significant (*F*(1,47) = 14.7, *p* < 0.001, partial *η*^2^ = 0.24). The subsequent Holm *post hoc* tests showed that RTs were faster (compared with all other conditions) when participants received We-information and the movements were the same (all *p*s ≤ 0.013). Moreover, if participants received the We-information but the movements were different, they were significantly faster compared with when they did not receive the We-information (*p*s ≤ 0.013). Finally, RTs for same and different configuration did not differ from each other if participants did not receive We-information (*p* = 0.691).

For the analysis on asynchronies, neither the main effect of We Information (*F*(1,23) = 1.8, *p* = 0.188), the main effect of configuration (*F*(1,23) = 0.4, *p* = 0.528), nor the interaction (*F*(1,23) = 0.3, *p* = 0.586) was significant.

#### (ii) Inclusion of other in self scale

A 2 × 2 ANOVA with factors time (pretest versus post-test) and agent (robot versus partner) revealed a significant main effect of time (*F*(1,47) = 56.9, *p* < 0.001, partial *η*^2^ = 0.55), reflecting larger self–other overlap (figure 2) during post-test and a significant main effect of agent (*F*(1,47) = 17.1, *p* < 0.001, partial *η*^2^ = 0.27) reflecting larger self**–**other overlap for partner, as well as a significant interaction (*F*(1,47) = 4.5, *p* = 0.038, partial *η*^2^ = 0.09). The *post hoc* tests for interaction revealed that all averages were significantly different from each other (all *p*s < 0.001, besides between post-test robot and post-test partner: *p* = 0.043), except for the difference between post-test with robot and pretest with partner (*p* = 0.082). It shows that the interaction was driven by higher initial overlap with the human partner and subsequent higher increase of  overlap for robot (average in pretest = 4.2 and post-test = 9.6), than for partner (average in pretest = 7.9 and post-test = 11.5).

### (b) EEG results

#### (i) Neural processes involved in processing of We-information

To investigate neural processes involved in processing and subsequent use of the We-information, we performed analyses time-locked to the presentation of the cue. We first contrasted trials in which We-information was present with those in which the We-information was absent, i.e. the cue was uninformative (contrast 1). Second, to determine whether the type of We-information affected the ERPs of interest, we additionally contrasted trials in which We-information indicated that the movements would be the same versus different (contrast 2, nested within contrast 1). For lateral posterior components, we included hemisphere as a factor in the analysis.

At the lateral posterior ROI, we investigated whether presentation of We-information affects the **P1**, **N1** and **P2p** components (figure 3). First, we performed repeated-measures 2 × 2 ANOVAs with factors We-information (present versus absent) and hemisphere (left versus right) for each of these components. We found that the main effect of We-information was significant for all three of them: P1 (*F*(1,47) = 26.1, *p* < 0.001, partial *η*^2^ = 0.36), N1 (*F*(1,47) = 39.0, *p* < 0.001, partial *η*^2^ = 0.45) and P2p (*F*(1,47) = 17.8, *p* < 0.001, partial *η*^2^ = 0.27). In all of these cases, the average amplitudes were larger in the absence of We-information. They were larger in the right hemisphere, although the effect of We-information was not modulated by hemisphere (see supplementary material, S1 for full results). For contrast 2 (same versus different configuration), we did not find evidence of any significant differences in any of these ERPs (supplementary material, S1).

At the frontal ROI, we performed paired *t-*tests comparing trials with and without the We-information (contrast 1) for the **frontal N2** and found that its amplitude was significantly larger in the absence of We-information (*t*(47) = 4.1, *p* < 0.001, *d* = 0.60). When comparing the type of We-information (contrast 2, same versus different configuration), we found that the amplitude of the N2 was significantly larger when We-information conveyed that the movements would be different rather than the same (*t*(47) = 3.0, *p* = 0.005, *d* = 0.43).

At the parietal ROI, we investigated the P3b component. By contrast with the earlier components, the **P3b** revealed larger amplitude when We-information was present (*t*(47) = 2.2, *p* = 0.032, *d* = 0.32). Contrast 2 did not yield a significant result (*t*(47) = 0.1, *p* = 0.958, *d* < 0.01).

At the central ROI, we investigated the **CNV**, which also revealed an increased amplitude when We-information was provided (*t*(47) = 3.0, *p* = 0.005, *d* = 0.43). Contrast 2 did not show a significant difference (*t*(47) = 0.7, *p* = 0.471, *d* = 0.11).

We did not observe the P600 component in our data.

#### (ii) The influence of We-information on recognition and processing of the target movement

To evaluate whether participants used We-information when processing the images of the target movements and preparing their response, we conducted analyses time-locked to the go-signal (figure 4). Because the go-signal provided complete information about what movement should be performed by each participant, instead of using two independent contrasts, we performed 2 × 2 analyses with factors We-information (present versus absent) and robot configuration (same versus different) because the second factor was not nested in the first one anymore. For the lateral posterior ROI, we also added hemisphere as a factor.

At the lateral posterior ROI, we once again investigated the **P1, N1 and P2p** (go-signal locked, this time, though) with the 2 × 2 × 2 repeated-measures ANOVA. For the P1, we found a significant main effect of We-information (*F*(1,47) = 7.4, *p* = 0.009, partial *η*^2^ = 0.14) reflecting increased amplitude in the absence of We-information. The main effect of hemisphere was also significant (*F*(1,47) = 14.0, *p* < 0.001, partial *η*^2^ = 0.23)—with higher amplitude in the right hemisphere. Importantly, three-way interaction was significant (*F*(1,47)  =  5.1,*p*  =  0.029, partial *η*^2^  =  0.10), reflecting that a reliable effect of We-information was present only in the left hemisphere. The main effect of We-information was also significant for the N1 (*F*(1,47) = 6.3, *p* = 0.016, partial *η*^2^ = 0.12) and P2p (*F*(1,47) = 31.9, *p* < 0.001, partial* η*^2^ = 0.40); full results of these analyses can be found in the supplementary material.

At the frontal ROI for the **N2**, there was a significant main effect for both We-information (*F*(1,47) = 15.8, *p* < 0.001, partial *η*^2^ = 0.25) and configuration (*F*(1,47) = 25.1, *p* < 0.001, partial *η*^2^ = 0.35). They reflected increased amplitude when We-information was absent, and when it indicated different movements, respectively. The interaction effect was not significant (*F*(1,47) = 0.1, *p* = 0.734, partial *η*^2^ < 0.01).

Finally, at the parietal site, for the **P3b**, we found a significant main effect of configuration (*F*(1,47) = 36.1, *p* < 0.001, partial *η*^2^ = 0.43), with higher amplitude for trials in which the movement was the same, while the main effect of We-information was not significant (*F*(1,47) = 0.8, *p* = 0.376, partial *η*^2^ = 0.02). The interaction effect was significant (*F*(1,47) = 10.9, *p* = 0.002, partial *η*^2^ = 0.19), reflecting the fact that the P3b was more strongly increased for the same positions if We-information was presented than in all other cases (all *ps* < 0.031). Moreover, if We-information was not present but the configuration was same then the P3b was significantly (*p* = 0.007 against We-info present & different) or marginally larger (*p* = 0.082 against We-info absent & different) than in the other conditions.

#### (iii) The influence of We-information on covert attention towards one's partner's side (lateralized ERPs)

To investigate allocation of covert spatial attention towards the partner's side, we investigated lateralized negative components (N1pc, N2pc and PCN) following presentation of the cue and the go-signal. Here, we present only the results of N2pc and PCN (figure 5). The results of the N1pc components are in S1 in the supplementary material.

For the cue-locked analyses, there was no clear N2pc peak, but instead we observed a sustained and increasing negativity until the appearance of the go-signal. Such effects are sometimes known under the general term of sustained PCN [44]. We performed analyses on the whole segment between the N1pc peak and the onset of the go-signal. We found that the mean amplitude was significantly more negative when We-information was absent (*t*(47) = 2.9, *p* = 0.005, *d* = 0.42), while the difference was not significant between two types of We-information (*t*(47) = 0.2, *p* = 0.858, *d* = 0.03).

For the go-signal-locked analyses, the 2 × 2 ANOVA for the N2pc component found a significant main effect of We-information (*F*(1,47) = 18.2, *p* < 0.001, partial *η*^2^ = 0.28), with larger amplitudes when it was present, and a significant main effect of configuration (*F*(1,47) = 64.6, *p* < 0.001, partial *η*^2^ = 0.58) with larger amplitude when movements were the same. The interaction was not significant (*F*(1,47) < 0.1, *p* = 0.899, partial *η*^2^ < 0.01).

## 4. Discussion

We conducted a dual-EEG study to investigate neural processes involved in coordination between two people jointly controlling a robot body. We were specifically interested in investigating neuro-cognitive mechanisms affected by presentation of the We-information, i.e. stimuli that convey information about properties at the group-level (in our case: the type of relationship between actions of two people), while being uninformative at the level of individuals (the specific movements to be performed by each individual) and irrelevant to the task itself. We were also interested in evaluating how joint control over the robot with another person affects integration of both the robot and the partner into one's self representation.

First, we found that providing We-information made participants faster. This is surprising, because possessing We-information is task-irrelevant and does not reduce uncertainty about one's own individual response. While the main effect of We-information replicated previous results [17,22], we also found a significant interaction effect that was different from previous studies. Kourtis *et al.* [17] investigated We-information in an interpersonal context of two people forming joint positions together, and found that the type of configuration (same versus different) did not have any effect on the RTs. By contrast, in [22], which was a behavioural version of the current study, reaction times were facilitated only when We-information indicated that the movements were going to be the same, and not when they were supposed to be different. The authors proposed that this interaction might be driven by automatic holistic processing of images of human bodies [45,46]. While the participants’ task is limited to focusing exclusively on their side of the robot, they might also automatically pay attention to the partner's side, leading to incongruency effects if two sides differ. Importantly, in contrast to [22], in the current study We-information accelerated RTs even when the robot's arm positions were different from each other, although this effect was smaller than when they were the same. A possible reason for this difference is that in [22], participants swapped places after every block, forcing frequent swapping of their attention between the two sides of the robot image. By contrast, in the current study they swapped places only once, making this readjustment much easier. This suggests that the influence of We-information on RTs might be driven by reduction of ***perceptual*** uncertainty about the incoming visual stimulus, even though task-relevant uncertainty remained constant across conditions (there was still the same 50% uncertainty about which button participant would need to press). However, this mechanism cannot be used to explain the results from the study by Kourtis *et al.* [17]—in which the target joint configuration of hand positions between two participants was never visually presented, and hence the perceptual uncertainty could not be reduced—suggesting that while it likely contributed in our study, it is probably not the only mechanism at play when people process We-information. The other mechanisms might be more directly related to processing of social information, as reported in studies on ingroup bias and prioritization of information about ‘we’ [47–49].

Similar to the behavioural study [22], and in contrast to [17], we did not find evidence that response asynchronies were modulated by We-information. This was probably owing to the fact that the current study was much easier, and the RT effects much smaller (up to 20 ms differences, as compared to over 100 ms differences there) leaving no space for the effect in asynchronies to emerge (similar to [22]).

Finally, we replicated a previous finding showing that joint control over a robot body leads to increased reported self–other overlap with both the robot and the task partner. However, in contrast to the previous study [22] in which this effect was similar for both robot and partner, in the current experiment this gain was much larger for the robot than the partner. This might be because pretest measures were taken after the lengthy procedure of preparing the EEG set-up, during which participants could interact and familiarize themselves with each other—leading to the relatively increased ratings of self–other overlap at the start of the task. Still, performing the joint control task elevated this overlap even further. This suggests that tasks involving embodied joint control might be useful to increase people's interpersonal closeness, as has been reported for other forms of joint action tasks [50,51]. In regard to the reported overlap between the robot and the self, our result suggests that even a brief period of control over the robot, even when it is shared, can lead to a form of incorporation of the robot into one's self-representation [22].

### (a) Neural processes involved in processing of the We-information

We found that presentation of We-information influenced a number of neural processes, as reflected by modulation of several ERPs: starting from early perceptual components P1, N1, N2pc through to posterior P2, frontal N2, then P3b, and finally modulating preparatory activity indexed by the CNV. However, while the majority of them were affected by the presence of We-information, only the N2 and the N2pc were sensitive to the type of We-information that was used (same versus other configuration).

The visual P1 and N1 primarily reflect early stages of visual input processing [42,52]. P1 is mostly driven by bottom-up processing, and as such reflects mostly low-level properties of seen stimuli. However, there is also evidence that it can be affected by top-down modulations [53–55]. In regard to the N1, this component has been canonically interpreted as also reflecting top-down processes on early stages of visual perception [56]. In our study, both of these components showed increased amplitude when the cue did not provide We-information. One plausible interpretation is that these effects reflect stimulus-driven differences because the informative and non-informative cues used different symbols (question mark versus in/equality sign). Alternatively, the effects may reflect top-down attentional expectations, with participants treating We-informative cues as more useful (because they can reduce the *perceptual* uncertainty), while non-informative cues are considered as less informative distractors. The influence of task-related prior expectations on these two components has been previously observed [57], making this interpretation plausible. However, future research involving stimuli arbitrarily associated with different types of We-information (similar to [58]) should resolve the question of which of these interpretations is correct.

At a slightly later stage, we observed modulation of the P2p component, similarly with higher amplitude in trials without the We-information. P2p has been frequently associated with processing of information about numerosity, specifically demonstrating higher amplitude when participants process stimuli involving larger numbers of objects [38,59]. While in our case, such stimuli were not displayed, the presence of We-information allowed a reduction in the number of the subsequent possible robot target positions from 4 to 2, so the reduction of its amplitude might reflect limiting the hypothesis space of options that one might ***observe*** afterwards.

At the frontal ROI, we observed modulation of the N2 component. The N2 effect was special because it was modulated not only by the presence of We-information, but also by its type. It was larger when We-information was not provided, but also when We-information indicated that participants would perform different movements rather than the same. Previous research found that N2 can reflect the magnitude of conflict between different potential response alternatives [60], although at the cognitive rather than motor level [25]. This interpretation is consistent with our findings. Receiving We-information allows one to reduce by half the ***perceptual*** uncertainty about the identity of the subsequent robot movements—the same sort of help that, as we postulate, is also reflected by the P2p. However, in contrast to the effect observed in the P2p, the representation indexed by the N2 appears to be more fine-grained and also takes into account the increased difficulty associated with processing of the robot's silhouette when the movements performed by each participant differ.

The pattern in which We-information leads to lower amplitude becomes reversed only at later stages of processing. We observed such reversal at the P3b component. The classical interpretation of the P3b treats it as an index of contextual updating [61], which is often interpreted as updating of the content of working memory [26,42]. Other theories underline the involvement of this component in decision making [62] and response preparation [63]. In our study, the increased amplitude in trials involving We-information might reflect increased working memory load, reflecting conscious processing of information conveyed by the We-informative cue and preparation of one's perceptual system to observe the target. However, this process does not reflect the expected difficulty of this task, as P3b is not sensitive to the form of We-information.

In the current study, we did not replicate the effect of We-information on the P600 component, which was reported by Kourtis *et al.* [17]. In fact, we did not observe a distinctive P600 at all. The most likely reason for the absence of this component is that We-information was presented alone in our study, whereas Kourtis *et al.* [17] presented it simultaneously with individual-level information. Consequently, the pattern of ERPs in our data was much less complex, showing fewer positive and negative deflections following the presentation of We-information. This replication failure indicates that the P600 is not a universal marker for processing We-information. Instead, its appearance is contingent on the task. Specifically, the P600 might indicate the processing of We-information only when presented together with individual-level information. Future studies are needed to validate this hypothesis.

Finally, corroborating previous findings from [17], we found that processing of We-information that was presented as a cue led to increase in the amplitude of the late CNV. CNV is a negative wave observed at the frontal–central electrodes before a target stimulus appears, thereby indicating processes related to expectancy [20,64]. We observed a sustained increase in its amplitude arising more than 500 ms before the onset of the go signal in trials with We-information. The CNV reflects anticipatory processes that take place before the second stimulus in a sequence of two—especially, but not only, when presentation of the second stimulus requires a response [65]. Current evidence suggests that these anticipatory processes involve both cognitive and motor preparatory processes [66]. Within the domain of social cognition, a previous study [67] found that CNV is also increased when two actions are prepared in parallel by one or two people, which raises an interesting possibility that the effect observed in our study might also reflect a form of co-representation of a partner's contribution.

### (b) The influence of We-information on recognition and processing of the target movement

As shown by the results from the previous section, processing and representing We-information leads to various forms of preparatory neural activity. Does this preparatory activity influence processing of the individual-level information and subsequent action execution? Our results, as shown by ERP analyses time-locked to the go signal, suggest that it does, and that it happens at several stages of cognition.

First, we found that if the cue involved We-information then it affected the amplitude of the lateral posterior P1, N1 and P2p, locked to the go-signal. The P1 was, again, increased if We-information was absent, while the N1 was lower. Importantly, in contrast to the cue-locked effect, these amplitude modulations cannot be explained by differences in low-level visual properties of the stimuli. The increased P1 might hence reflect increased sensory gain when participants process the target movement, while having no prior knowledge from We-information. In such cases, they might expect their task to be more demanding and hence they enhance their attention [68]. By contrast with P1, N1 is more sensitive to top-down effects. Among them are category- and object-specific expectations [69]. Its increased amplitude might hence reflect a match of the perceived image with sensory expectations formed after processing of the We-information. Finally, the increased amplitude of the P2p might once again reflect selection from among a larger number of potential options.

The frontal N2 is, again, the earliest component that is sensitive to both whether We-information was presented and to the movement configuration. Importantly, these two factors did not interact with each other. As discussed above, the N2 is most often interpreted as reflecting conflict between competing response alternatives, although at the cognitive rather than motor level. This is consistent with our results, where We-information did not reduce uncertainty about participants’ response (each was still equally likely), but it did reduce perceptual uncertainty about which robot full-body positions can appear and will be observed; hence, the amplitude of the N2 was smaller if We-information was provided. Second, it can also explain why the N2 was smaller when the robot movement configuration was same. As shown by behavioural results from the current and a previous study [22], participants display slower RTs when robot configuration is different. This slowing of RTs is probably caused by the interference from visual processing of the partner's side of the robot body. In cases when the movements of both arms are the same, this interference disappears, making it easier to detect which movement one should perform, leading to a reduced N2. Finally, the fact that these two factors did not interact means that the facilitation reflected by the N2 does not depend on We-information.

Finally, the P3b component following presentation of the go signal showed the main effect of configuration, as well as a significant interaction between configuration and We-information. First, it was increased in trials when the configuration was same (both arms up or both arms down). However, its amplitude was especially elevated in trials with the same configuration in which participants received We-information. By contrast, when We-information specified that the configuration was different, then the P3b was not significantly higher than in no We-information conditions. In other words, P3b was increased when the configuration was same. This was especially strong if participants knew about it in advance, and in such cases, visual inspection suggests that the P3b was followed by increased late positive potential (LPP). The LPP is generally understood to reflect sustained attentional engagement for motivationally relevant stimuli, and especially for stimuli that are emotionally engaging [70–72]. This might reflect the fact that if the robot performs symmetric movements, they might be more visually appealing and rewarding to participants [73–76]. If this interpretation is correct, then the observed pattern of results might indicate that performance of the same movements is especially rewarding to the observer when they are primed with relevant We-information.

### (c) The influence of We-information on allocation of covert attention to one's partner's side

The negative posterior contralateral components reflect the magnitude of covert attention paid to the contralateral side. We investigated this both following presentation of the cue and the target. After the cue, we observed a sustained PCN reflecting prolonged covert attention towards the side of the partner. Importantly, the amplitude of this sustained PCN was larger in trials without the We-information, suggesting that when it was present they focused more on processing it, while when it was absent they started covertly monitoring their partner more, up until the arrival of the fully informative go-signal. This heightened monitoring of the partner might reflect the shift of focus towards a potentially more informative source of information for interpersonal coordination.

Interestingly, the N2pc following the go-signal displayed the opposite pattern—its amplitude was larger when We-information was provided. We propose that an informative cue led to increased N2pc because when participants already had prior information, they could focus more of their covert attention on monitoring and coordinating with their partner, while if this information was absent, they had to focus more strongly on processing the centrally presented image of the target movement. The N2pc was also larger when the movements were the same than different (regardless of the presence or absence of We-information). This means that participants paid more attention to the partner's side when the movements were the same. This probably reflects the fact that when movements were different, participants had to take extra effort to block processing of stimuli from the partner's side and focus on theirs. On the other hand, if the movements performed by both robot arms were the same, they could pay attention to either side of the robot to learn which movement they should perform. The results of the N1pc components, reported in S1 in the supplementary material, were also consistent with these interpretations. Overall, these results show that participants can dynamically allocate their covert attention to the side of their partner, depending on the presence of We-information. They also contribute to the recently increased interest in the study of covert social attention [27].

### (d) Limitations and future direction

The current study shows that processing of We-information can influence a wide range of cognitive processes. However, what remains an open question is whether the effects observed in our study are social in nature, or only reflect domain-general mechanisms of uncertainty reduction. A very similar debate began in 2010 about the process of automatic visual perspective-taking, where one side of the debate argued for its social nature [77,78], while the other reasoned that it can be explained through non-social domain-general mechanisms [79,80]. This spawned a large number of subsequent studies, but so far no consensus has been achieved. Our current results also do not allow us to provide a definite answer to this question with respect to We-information. The fact that We-information used in our study was presented to participants in the social and embodied context (joint control over a humanoid robot) shows that it can be useful and effective in social context; however, it does not mean that the underlying mechanisms have to be such. To fully test the social aspects of the task, one would need to manipulate three aspects systematically: first, and most importantly, whether the task is performed with another person, a non-human social agent, a non-social agent or individually. Second, our task involved joint control over a humanoid robot body and, as such, it involved both a social and an embodied aspect. This could be contrasted with joint control over a tool or a non-social robot, such as an industrial robot arm, where these factors are absent. Third, our task involved presenting a picture of a robot as a target, to make the task model a potential real-life situation in which users need to jointly coordinate their robotic body's movements. However, using symbolic non-social targets might provide a way to probe the extent to which the effects we observed are related to processing of bodily images. Future research should explore these possibilities to determine which of our results are driven by social mechanisms in the brain. Future studies can also use more advanced techniques of brain imaging and data analysis (combined EEG and fMRI recordings, allowing source localization) that could help to determine the specific neural underpinnings of the processing of We-information.

## 5. Conclusions

We investigated which neurocognitive processes are affected by the presentation of We-information during joint control over a single robot body. Our study shows that We-information can have a much larger impact than has been previously reported and that it affects a cascade of cognitive processes, from early perception, through response selection, working memory updating and response preparation.

Previous research has demonstrated that processing of self-related information can have a profound influence on cognition [81–83], including within the context of one's group membership [47,84]. Our study shows that processing information about the We—as specifically shown through an example of joint robot teleoperation—can demonstrate a similarly widespread influence. However, one outstanding question is to what extent the effects observed in our study reflect only the employment of domain-general cognitive mechanisms to address social cognitive needs, and to what extent they might reflect a wider social cognitive process, such as entering a ‘We-mode’ with another person [9,10] and potentially also with the robotic body. This would indicate a true marker of simultaneously embodied and socially extended self.

Overall, our study shows that processing We-information can exert a widespread influence on neural processes by affecting early perceptual mechanisms, as well as later stages of action preparation and execution, demonstrating its influence on multiple levels of cognition. Beyond their theoretical contribution to the study of We-representations, these findings have applied implications for the design of cooperative robotic systems, suggesting that providing group-level relational cues may facilitate coordination and anticipation during shared control. They might help to guide the design of robotic systems through which people work jointly together. Using We-information might also become useful in neurorehabilitation and neuroprosthetics. For example, it might be useful when learning how to coordinate between a healthy limb and a prosthetic limb controlled by AI (in everyday use) or another person (during rehabilitation).

## Supplementary Material



## Data Availability

Behavioural data, analysis scripts and materials are available in the following repository [85]. EEG data are available upon request from the corresponding authors. Supplementary material is available online [86].

## Theme

One contribution of 17 to a discussion meeting issue ‘From being to identity: analysis and synthesis of the self’.
