# Supplementary material for: Two minds, one body: neural correlates of processing of We-information during shared control over a humanoid robot
Source: Philos Trans R Soc Lond B Biol Sci. 2026 Nov 10;381(1962):20250163. doi: 10.1098/rstb.2025.0163 (PMC13750424; doi:10.1098/rstb.2025.0163)
Supplement: Supplementary file 1 [file rstb.2025.0163.zip › rstb.2025.0163/rstb20250163_si_001.pdf]

Supplementary Materials S1

Target article: Woźniak, Hahxi, De Tommaso, Wykowska (2026). **Two Minds, One Body: Neural correlates of processing of we-information during shared control over a humanoid robot**. *Proceedings of the Royal Society B: Biological Sciences*

Full EEG analyses

These supplementary materials report full results of the analyses reported in the main text but were removed due to length requirements of the manuscripts and the need for readability. It follows the order of presentation of the ERPs from the manuscript.

CUE-LOCKED ANALYSES

Lateral posterior ROIs.

At the posterior lateral ROI, we investigated whether presentation of We-information affects the P1, N1 and P2p components. First, we performed repeated-measures 2x2 ANOVAs with factors We-Information (present vs absent) and hemisphere (left vs right) for each of these components and found that presence of We-information led to reduction of the amplitude of all three of them.

P1.

For P1 there was the main effect of We-information (F(1,47)=26.1, p<0.001, partial η^2=0.36) and hemisphere (F(1,47)=27.8, p<0.001, partial η^2=0.37), while the interaction was not significant (F(1,47)=2.3, p=0.134, partial η^2=0.05).

***Table**. 2x2 ANOVA: We-information (present vs. absent) x Hemisphere (left vs. right)*

We-information present					
	F value	*Num DF*	*Def DF*	*Pr>F (p)*	*Eta2*
WeNo	26.0507	1.0000	47.0000	0.0000	0.3566
ROILeftRight	27.8138	1.0000	47.0000	0.0000	0.3718
WeNo:ROILeftRight	2.3232	1.0000	47.0000	0.1342	0.0471

Averages	
PRECUE_We Info_ROILeft	1.96
PRECUE_No Info_ROILeft	2.25
PRECUE_We Info_ROIRight	3.00
PRECUE_No Info_ROIRight	3.44

Table. 2x2 ANOVA: Configuration (same vs. different) x Hemisphere (left vs. right)

	F value	*Num DF*	*Def DF*	*Pr>F (p)*	*Eta2*
WeNo	0.1052	1.0000	47.0000	0.7471	0.0022
ROILeftRight	28.1264	1.0000	47.0000	0.0000	0.3744
WeNo:ROILeftRight	0.9291	1.0000	47.0000	0.3400	0.0194

Averages	
PRECUE_We Info Same_ROILeft	1.95
PRECUE_We Info Different_ROILeft	2.04
PRECUE_We Info Same_ROIRight	3.02
PRECUE_We Info Different_ROIRight	2.93

N1.

For N1 there was a significant main effect of We-information (F(1,47)=39.0, $p<0.001$, partial $\eta^2=0.45$), while the main effect of hemisphere (F(1,47)=0.7, $p=0.732$, partial $\eta^2<0.01$) and the interaction effect (F(1,47)=2.4, $p=0.125$, partial $\eta^2=0.05$) were not significant.

Table. 2x2 ANOVA: We-information (present vs. absent) x Hemisphere (left vs. right)

	F value	*Num DF*	*Def DF*	*Pr>F (p)*	*Eta2*
WeNo	38.9572	1.0000	47.0000	0.0000	0.4532
ROILeftRight	0.1188	1.0000	47.0000	0.7319	0.0025
WeNo:ROILeftRight	2.4452	1.0000	47.0000	0.1246	0.0495

Averages	
PRECUE_We Info_ROILeft	-1.22
PRECUE_No Info_ROILeft	-1.67
PRECUE_We Info_ROIRight	-1.00
PRECUE_No Info_ROIRight	-1.71
Sub_Num	1.16

Table. 2x2 ANOVA: Configuration (same vs. different) x Hemisphere (left vs. right)

	F value	*Num DF*	*Def DF*	*Pr>F (p)*	*Eta2*
WeNo	2.4792	1.0000	47.0000	0.1221	0.0501
ROILeftRight	0.7867	1.0000	47.0000	0.3796	0.0165
WeNo:ROILeftRight	6.1335	1.0000	47.0000	0.0169	0.1154

Averages	
PRECUE_We Info Same_ROILeft	-1.23
PRECUE_We Info Different_ROILeft	-1.22
PRECUE_We Info Same_ROIRight	-8.30
PRECUE_We Info Different_ROIRight	-1.1

P2p.

For P2p the main effect of We-information ($F(1,47)=17.8$, $p<0.001$, partial $\eta^2=0.27$) and hemisphere ($F(1,47)=6.7$, $p=0.013$, partial $\eta^2=0.12$) were significant but the interaction was not ($F(1,47)=1.3$, $p=0.266$, partial $\eta^2=0.03$).

Table. *2x2 ANOVA: We-information (present vs. absent) x Hemisphere (left vs. right)*

	F value	*Num DF*	*Def DF*	*Pr>F (p)*	*Eta2*
WeNo	17.7572	1.0000	47.0000	0.0001	0.2742
ROILeftRight	6.6802	1.0000	47.0000	0.0129	0.1244
WeNo:ROILeftRight	1.2650	1.0000	47.0000	0.2664	0.0262

Averages	
PRECUE_We Info_ROILeft	1.02
PRECUE_No Info_ROILeft	1.58
PRECUE_We Info_ROIRight	1.62
PRECUE No Info ROIRight	2.02

Table. *2x2 ANOVA: Configuration (same vs. different) x Hemisphere (left vs. right)*

	F value	*Num DF*	*Def DF*	*Pr>F (p)*	*Eta2*
WeNo	6.7383	1.0000	47.0000	0.0126	0.1254
ROILeftRight	6.6127	1.0000	47.0000	0.0133	0.1233
WeNo:ROILeftRight	2.8389	1.0000	47.0000	0.0986	0.0570

Averages	
PRECUE_We Info Same_ROILeft	8.57
PRECUE_We Info Different_ROILeft	1.20
PRECUE_We Info Same_ROIRight	1.56
PRECUE_We Info Different_ROIRight	1.69

In all of these cases the average amplitudes were larger in the absence of We-information, and they were larger in the right hemisphere, although the effect of We-information was not modulated by hemisphere. For contrast 2 (same vs., different configuration) we did not find evidence of any significant differences in any ERP.

Frontal ROI.

N2.

At the frontal ROI we performed paired t-tests comparing trials with and without the We-information (contrast 1) for the frontal N2 and found that its amplitude was significantly larger in the absence of We-information ($t(47)=4.1$, $p<0.001$, $d=0.60$). When comparing the type of We-information (contrast 2, same vs. different configuration) we found that the amplitude of the N2 was significantly larger when We-information conveyed that the movements would be different rather than the same ($t(47)=3.0$, $p=0.005$, $d=0.43$).

Table. *T-test: We-information (present vs. absent)*

We Info	-0.8339891295455442
No Info	-1.2344952899999166

T-statistic	4.1405
df	47
p-value	0.0001
Cohen'd	0.598

Table. *T-test: Configuration (same vs. different)*

We Info	-0.6400407536199968
No Info	-1.026448947215569

T-statistic	3.5010
df	47
p-value	0.0010
Cohen'd	0.505

Parietal ROI.

P3b.

At the parietal ROI we investigated the P3b component. In contrast to the earlier components the **P3b** revealed larger amplitude when We-information was present ($t(47)=2.2$, $p=0.032$, $d=0.32$). Contrast 2 did not yield a significant result ($t(47)=0.1$, $p=0.958$, $d<0.01$).

Table. *T-test: We-information (present vs. absent)*

We Info	-0.401733153022227
No Info	0.5604991391260414

T-statistic	2.2115
df	47
p-value	0.0319
Cohen'd	0.319

Table. *T-test: Configuration (same vs. different)*

We Info	-0.39964007388557415
No Info	-0.40347463902511677

T-statistic	0.0532
df	47
p-value	0.9578
Cohen'd	0.008

Central ROI.

CNV.

At the central ROI we investigated the **CNV**, which also revealed an increased amplitude when We-information was provided ($t(47)=3.0$, $p=0.005$, $d=0.43$). Contrast 2 did not show a significant difference ($t(47)=0.7$, $p=0.471$, $d=0.11$).

Table. *T-test: We-information (present vs. absent)*

We Info	-1.7410759551549426
No Info	-1.4884046907700212

T-statistic	-2.9678
df	47
p-value	0.0047
Cohen'd	-0.428

***Table**. T-test: Configuration (same vs. different)*

We Info	-1.7877256783682682
No Info	-1.693446319453341

T-statistic	-0.7269
df	47
p-value	0.4709
Cohen'd	-0.105

GO-SIGNAL-LOCKED ANALYSES

Lateral posterior ROIs.

P1. For the P1 we found a significant main effect of We-information (F(1,47)=7.4, $p<0.001$, partial $\eta^2=0.14$) reflecting increased amplitude in the absence of We-information. The main effect of hemisphere was also significant (F(1,47)=14.0, $p<0.001$, partial $\eta^2=0.23$) – with higher amplitude in the right hemisphere. Importantly, 3-way interaction was significant (F(1,47)=5.1, $p=0.029$, partial $\eta^2=0.10$) reflecting that a reliable effect of We-information was present only in the left hemisphere.

***Table**. 2x2x2 ANOVA: We-information (present vs. absent) x Configuration (same vs. different) x Hemisphere (left vs. right)*

	F value	*Num DF*	*Def DF*	*Pr>F (p)*	*Eta2*
WeNo	7.4441	1.0000	47.0000	0.0089	0.1367
SameDiff	1.0996	1.0000	47.0000	0.2997	0.0229
ROILeftRight	13.9633	1.0000	47.0000	0.0005	0.2290
WeNo*: SameDiff*	0.3952	1.0000	47.0000	0.5326	0.0083
WeNo:ROILeftRight	0.5979	1.0000	47.0000	0.4433	0.0126
SameDiff:ROILeftRight	4.2896	1.0000	47.0000	0.0439	0.0836
WeNo: SameDiff:ROILeftRight	5.0618	1.0000	47.0000	0.0292	0.0972

Averages	
GO_We Same_ROILeft	3.46
GO_No Same_ROILeft	3.65
GO_We Different_ROILeft	3.24
GO_No Different_ROILeft	3.54
GO_We Same_ROIRight	5.06
GO_No Same_ROIRight	5.35
GO_We Different_ROIRight	5.22
GO_No Different_ROIRight	5.23

N1. For the N1 only the main effect of We-information was significant (F(1,47)=6.3, *p*=0.016, partial η^2=0.12), but this time the amplitude was higher in trials with We-information.

Table*. 2x2x2 ANOVA: We-information (present vs. absent) x Configuration (same vs. different) x Hemisphere (left vs. right)*

	F value	*Num DF*	*Def DF*	*Pr>F (p)*	*Eta2*
WeNo	6.2674	1.0000	47.0000	0.0158	0.1177
SameDiff	0.6730	1.0000	47.0000	0.4162	0.0141
ROILeftRight	0.0025	1.0000	47.0000	0.9600	0.0001
WeNo*: SameDiff*	0.0067	1.0000	47.0000	0.9351	0.0001
WeNo:ROILeftRight	1.1499	1.0000	47.0000	0.2890	0.0239
SameDiff:ROILeftRight	3.8933	1.0000	47.0000	0.0544	0.0765
WeNo: SameDiff:ROILeftRight	0.0004	1.0000	47.0000	0.9842	0.0000

Averages	
GO_We Same_ROILeft	-4.79
GO_No Same_ROILeft	-3.24
GO_We Different_ROILeft	-5.21
GO_No Different_ROILeft	-3.79
GO_We Same_ROIRight	-6.64
GO_No Same_ROIRight	-4.07
GO_We Different_ROIRight	-4.86
GO_No Different_ROIRight	-2.39

P2p. For the P2p the main effect of We-information was significant (F(1,47)=31.9, p<0.001, partial η2=0.40) again showing higher amplitude when there was no We-information. Besides it, only the main effect of hemisphere was significant (F(1,47)=10.5, p=0.002, partial η2=0.18) – with larger amplitude on the right.

Table*. 2x2x2 ANOVA: We-information (present vs. absent) x Configuration (same vs. different) x Hemisphere (left vs. right)*

	F value	*Num DF*	*Def DF*	*Pr>F (p)*	*Eta2*
WeNo	31.9694	1.0000	47.0000	0.0000	0.4048
SameDiff	2.4353	1.0000	47.0000	0.1253	0.0493
ROILeftRight	10.5236	1.0000	47.0000	0.0022	0.1829
WeNo*: SameDiff*	1.8025	1.0000	47.0000	0.1859	0.0369
WeNo:ROILeftRight	1.5165	1.0000	47.0000	0.2243	0.0313

SameDiff:ROILeftRight	0.8033	1.0000	47.0000	0.3747	0.0168
WeNo: SameDiff:ROILeftRight	2.8234	1.0000	47.0000	0.0995	0.0567

Averages	
GO_We Same_ROILeft	4.06
GO_No Same_ROILeft	4.44
GO_We Different_ROILeft	4.29
GO_No Different_ROILeft	4.64
GO_We Same_ROIRight	5.87
GO_No Same_ROIRight	6.51
GO_We Different_ROIRight	6.10
GO_No Different_ROIRight	6.43

Frontal ROI.

N2.

***Table**. 2x2 ANOVA: We-information (present vs. absent) x Configuration (same vs. different)*

	F value	*Num DF*	*Def DF*	*Pr>F (p)*	*Eta2*
WeNo	22.3939	1.0000	47.0000	0.0000	0.3227
SameDiff	0.3483	1.0000	47.0000	0.5579	0.0074
ROILeftRight	0.1643	1.0000	47.0000	0.6871	0.0035

We Same	-2.9450104711878184
No Same	-3.253367227333616
We Different	-2.973414226891652
No Different	-3.330547605699627

Parietal ROI.

P3b.

At the parietal ROI we investigated the P3b component. In contrast to the earlier components the **P3b** revealed larger amplitude when We-information was present ($t(47)=2.2$, $p=0.032$, $d=0.32$). Contrast 2 did not yield a significant result ($t(47)=0.1$, $p=0.958$, $d<0.01$).

***Table**. 2x2 ANOVA: We-information (present vs. absent) x Configuration (same vs. different)*

	F value	*Num DF*	*Def DF*	*Pr>F (p)*	*Eta2*
WeNo	0.7985	1.0000	47.0000	0.3761	0.0167
SameDiff	36.1419	1.0000	47.0000	0.0000	0.4347
ROILeftRight	10.8618	1.0000	47.0000	0.0019	0.1877

We Same	3.517238813394989
No Same	3.2698575872527313
We Different	2.982914396588048
No Different	3.1127739281069613

LATERALIZED ERP ANALYSES

Cue-locked N1pc. We found that the amplitude of the N1pc ($t(47)=4.1$, $p<0.001$, $d=0.59$) was larger when We-information was absent. Moreover, when We-information was present the N1pc was significantly larger when it indicated that the movements are different than the same ($t(47)=2.3$, $p=0.027$, $d=0.33$).

Cue-locked PCN. For the cue-locked analyses there was no clear N2pc peak, but instead we observed a sustained and increasing negativity until the appearance of the go-signal. Such effects are sometimes known under the general term of PCN (posterior contralateral negativity). We performed analyses on the whole segment between the N1pc peak and the onset of the go-signal. We found that the mean amplitude was significantly more negative when We-information was absent ($t(47)=2.9$, $p=0.005$, $d=0.42$), while the difference was not significant between two types of We-information ($t(47)=0.2$, $p=0.858$, $d=0.03$).

Go-locked N1pc. For the go-signal-locked analyses we performed a 2x2 ANOVA for each component. For the N1pc there was a significant main effect of We-information ($F(1,47)=13.2$, $p<0.001$, partial $\eta 2=0.2$) reflecting larger amplitude if it was present. The main effect of Configuration was not significant ($F(1,47)=1.4$, $p=0.238$, partial $\eta 2=0.03$). The interaction was significant ($F(1,47)=11.8$, $p=0.001$, partial $\eta 2=0.20$) what reflected that the amplitude was much higher for We-information trials with Same configuration than in the remaining ones (all $ps<0.022$, Holm corrected). Besides, only We-information different had a significantly higher amplitude than No-information same ($p=0.034$).

Go-locked N2pc. For the N2pc we found a significant main effect of We-information ($F(1,47)=13.7$, $p<0.001$, partial $\eta 2=0.23$) with larger amplitudes when it was present, and a significant main effect of Configuration ($F(1,47)=35.7$, $p<0.001$, partial $\eta 2=0.43$) with larger amplitude when movements were the same. The interaction was not significant ($F(1,47)=0.2$, $p=0.625$, partial $\eta 2<0.01$).
